# Metabolomics Reveals Metabolic Alterations by Intrauterine Growth Restriction in the Fetal Rabbit Brain

**DOI:** 10.1371/journal.pone.0064545

**Published:** 2013-05-27

**Authors:** Erwin van Vliet, Elisenda Eixarch, Miriam Illa, Ariadna Arbat-Plana, Anna González-Tendero, Helena T. Hogberg, Liang Zhao, Thomas Hartung, Eduard Gratacos

**Affiliations:** 1 Department of Maternal-Fetal Medicine, Institut Clinic de Ginecologia, Obstetricia i Neonatologia (ICGON), Hospital Clinic and Institut d'Investigacions Biomediques August Pi i Sunyer (IDIBAPS), University of Barcelona, Barcelona, Spain; 2 Johns Hopkins University, Bloomberg School of Public Health, Department of Environmental Health Science, Baltimore, Maryland, United States of America; Hôpital Robert Debré, France

## Abstract

**Background:**

Intrauterine Growth Restriction (IUGR) due to placental insufficiency occurs in 5–10% of pregnancies and is a major risk factor for abnormal neurodevelopment. The perinatal diagnosis of IUGR related abnormal neurodevelopment represents a major challenge in fetal medicine. The development of clinical biomarkers is considered a promising approach, but requires the identification of biochemical/molecular alterations by IUGR in the fetal brain. This targeted metabolomics study in a rabbit IUGR model aimed to obtain mechanistic insight into the effects of IUGR on the fetal brain and identify metabolite candidates for biomarker development.

**Methodology/Principal Findings:**

At gestation day 25, IUGR was induced in two New Zealand rabbits by 40–50% uteroplacental vessel ligation in one horn and the contralateral horn was used as control. At day 30, fetuses were delivered by Cesarian section, weighed and brains collected for metabolomics analysis. Results showed that IUGR fetuses had a significantly lower birth and brain weight compared to controls. Metabolomics analysis using liquid chromatography-quadrupole time-of-flight mass spectrometry (LC-QTOF-MS) and database matching identified 78 metabolites. Comparison of metabolite intensities using a t-test demonstrated that 18 metabolites were significantly different between control and IUGR brain tissue, including neurotransmitters/peptides, amino acids, fatty acids, energy metabolism intermediates and oxidative stress metabolites. Principle component and hierarchical cluster analysis showed cluster formations that clearly separated control from IUGR brain tissue samples, revealing the potential to develop predictive biomarkers. Moreover birth weight and metabolite intensity correlations indicated that the extent of alterations was dependent on the severity of IUGR.

**Conclusions:**

IUGR leads to metabolic alterations in the fetal rabbit brain, involving neuronal viability, energy metabolism, amino acid levels, fatty acid profiles and oxidative stress mechanisms. Overall findings identified aspargine, ornithine, N-acetylaspartylglutamic acid, N-acetylaspartate and palmitoleic acid as potential metabolite candidates to develop clinical biomarkers for the perinatal diagnosis of IUGR related abnormal neurodevelopment.

## Introduction

Intrauterine Growth Restriction (IUGR) due to placental insufficiency occurs in 5–10% of gestations and is a major determinant of perinatal morbidity and mortality [1]. The compromised placental blood supply results in sustained hypoxemia and under-nutrition of the developing fetus [2], which can affect fetal programming of vital organs and an increased risk for disease later in life [3], [4]. The fetal brain was shown to be particularly vulnerable to prolonged IUGR conditions [5]–[6]. Clinical imaging studies demonstrated structural changes in the brain of IUGR infants including altered white and grey matter volumes [7]–[8], decreased levels of brain connectivity [9] and delayed cortical development [10]. Moreover, several clinical studies in neonates and infants who suffered IUGR showed both short and long-term neurodevelopmental delays and cognitive dysfunctions [11]–[12].

The early perinatal diagnosis of IUGR related abnormal neurodevelopment represents a major challenge in fetal medicine. At present abnormal neurodevelopment is primarily diagnosed later in the child's life and persists until late childhood and adolescence [13]–[14]. The ability to diagnose abnormal neurodevelopment during perinatal life would allow interventions during the critical window of opportunity of the first two years of life, when brain reorganization is particularly active and interventions have shown to be effective in reverting the effects of adverse fetal conditions [15]. The development of clinical biomarkers is considered a promising approach to predict and monitor abnormal neurodevelopment during perinatal life. Because the effects of IUGR on the fetal brain are subtle, the approach will likely involve the identification of sensitive biochemical and/or molecular imaging biomarkers [16]. The discovery and development of such biomarkers requires an improved understanding of the underlying molecular and biochemical mechanisms of IUGR related abnormal neurodevelopment.

Metabolomics is defined as the quantitative measurement of the dynamic metabolic response of living systems to genetic, physical, pathological or developmental factors [17]. The technology has emerged in numerous fields of research, including fetal medicine aiming to facilitate the understanding of fetal disease pathophysiology and discovery of predictive biomarkers [18]. Previous metabolomics studies have shown metabolic alterations in clinical samples including cord blood and urine samples of IUGR neonates [19]–[20]. Moreover, metabolic profiling studies in animal IUGR models (e.g. rat and pig) pointed out alterations in blood plasma, serum and jejunum metabolome as potential biomarkers for the diagnosis of IUGR and its effects on fetal programming [21]–[23]. To the best of our knowledge, the effects of IUGR on the fetal brain metabolome have not yet been characterized.

The aim of this targeted metabolomics study was to obtain more mechanistic insight into the effects of IUGR on the fetal brain and identify potential metabolite candidates for the development of clinical biomarkers for IUGR related abnormal neurodevelopment. The study was undertaken in a rabbit model of IUGR, which mimics placental insufficiency by the selective 40–50% ligature of uteroplacental vessels [24]. Despite general species differences, brain development in the rabbit is similar to that of humans, but within a more compressed time-frame [25]. As in humans, the temporal pattern of oligodendrocyte maturation, myelination and functional changes occur most rapidly during the perinatal period, starting several days before birth and continuing during the postnatal period. Therefore the rabbit provides a relevant and useful model to study perinatal processes of brain development. Previous studies in the rabbit IUGR model, showed that IUGR leads to impairment, hemodynamic adaptation, alterations in white and grey brain matter and neurobehavioral changes, as observed in IUGR infants [24], [26]. The findings of this targeted metabolomics study provide additional mechanistic insights into the effects of IUGR on the fetal brain and support the development of clinical biomarkers for the perinatal diagnosis or treatment of IUGR related abnormal neurodevelopment.

## Materials and Methods

### The Rabbit IUGR Model

All animal procedures in this study were approved by the Animal Experimental Ethics Committee of the University of Barcelona. Moreover animal handling and procedures were performed according the applicable regulations and guidelines of the Animal Experimental Ethics Committee of the University of Barcelona (Permit number: 2012/7684). The New Zealand pregnant rabbits were provided by a certified breeder and dams were housed for 1 week before surgery in separate cages on a reversed 12/12 hour light cycle, with free access to water and standard chow. IUGR was induced in two New Zealand pregnant rabbits at 25 days of gestation by uteroplacental vessels ligation as described in [24]. Briefly, after tocolysis and antibiotic prophylaxis administration, an abdominal midline laparotomy was performed under anaesthetic condition. Gestational sacs of both horns were identified and, in one uterine horn, 40–50% of the uteroplacental vessels of all gestational sacs were ligated, the contra lateral horn was used as control. After the procedure the abdomen was closed in two layers with a single suture of silk (3/0). Postoperative analgesia was administered and animals were again housed with free access to water and standard chow for 5 days until delivery and well-being was controlled each day. Cesarean section was performed at 30 days of gestation (term at 31 days) and nine living (five control and four IUGR) and seven stillborn (one control and six IUGR) fetuses were collected. After delivery, all living newborns were weighed and sacrificed by immediate decapitation. The fetal brains were removed, weighed and anterior hemisphere, posterior hemisphere, basal ganglia and brain stem regions were dissected on ice. The dissected brain tissues were snap frozen in liquid nitrogen and thereafter stored at −80°C for subsequent metabolomics analysis.

### Sample Preparation Procedures

The frozen brain tissue regions were weighted and transferred to an Eppendorf tube. For metabolite extraction (25 µL/mg of tissue) ice cold high purity Methanol/Water mixture v/v 70/30 (Sigma Aldrich, Spain) was added. The brain tissues were disrupted (on ice) using an ultrasound sonicator (Qsonica, CT, USA) for 3–4 minutes until no more cellular debris was visible in the tissue homogenate. To control for potential differences in tissue quantities the total protein content of the homogenates was quantified using a protein assay (BioRad). A standard volume of 250 µL containing 10 mg of tissue was transferred to a new Eppendorf tube, stored for 1 hour at −20°C and centrifuged at 16,000 rcf, 4°C for 10 min. Supernatants were collected, transferred to a new eppendorf tube and evaporated to dryness at room temperature in a speedvac concentrator (Thermo Scientific). The dried samples were reconstituted in 60 µL of 60% MeOH with 0.1% FA and clarified by centrifugation at 14,000 rcf, 4°C for 5 minutes. The clarified samples were transferred to plastic HPLC vials for LC-QTOF-MS measurements.

### Liquid Chromatography-quadrupole Time-of-flight Mass Spectrometry Measurements

The LC-QTOF-MS measurements were performed using an Agilent 1260 series LC binary pump and wellplate autosampler coupled to a 6520 accurate-mass Q-TOF LC-MS system equipped with a dual electrospray (ESI) ion source operated in negative-ion mode (Agilent Technologies, Santa Clara, CA). A Cogent Diamond Hydride ™ (MicroSol, Eatontown, NJ) aqueous normal phase (ANP) column (150×2.1 mm i.d., 4 µm particle size, 100 µm pore size) was used for separation of metabolites. The LC parameters were as follows: autosampler temperature, 4°C; injection volume, 5 µl; column temperature, 35°C; flow rate, 0.4 ml/min. The solvents and optimized gradient conditions for LC were: Solvent A, 50% methanol/50% water/0.05% formic acid; Solvent B, 90% acetonitrile with 5 mM ammonium acetate; elution gradient: 0 min–100% B; 20–25 min –40% B; post-run time for equilibration, 10 min in 100% B. A blank injection was run after every 3 samples. The optimized ESI Q-TOF parameters for MS experiments were: ion polarity, negative; gas temperature, 325°C; drying gas, 10 l/min; nebulizer pressure, 45 psig; capillary voltage, 4,000 V; fragmentor, 140 V; skimmer, 65 V; mass range, 70–1,100 m/z; acquisition rate, 1.5 spectra/s; instrument state, extended dynamic range (1,700 m/z, 2 GHz); Spectra were internally mass calibrated in real time by continuous infusion of a reference mass solution using an isocratic pump connected to a dual sprayer feeding into an electrospray ionization source. Data were acquired with MassHunter Acquisition software (Agilent Technologies, Santa Clara, CA).

### Data Processing and Metabolite Identification

Following LC-QTOF-MS data acquisition, the acquired raw data files were processed with Agilent MassHunter Qualitative Analysis software (version 5.0). Reproducibility of chromatograms was first inspected by overlaying the Total Ion Chromatograms (TICs) of all samples. Data files that showed extraneous peaks were excluded for further processing. To normalize the samples for differences in tissue quantities the OD values of the protein assay were integrated into the ion intensity quantification procedure. The normalization procedure was confirmed by a comparison of the total ion intensity of peaks in MS profiles. Initial, putative metabolite identification was achieved by searching the accurate m/z values of the peaks against an in-house built database derived from HMDB, KEGG, METLIN and other public databases. At the same time, the Extracted Ion Chromatograms (EICs) for these matched putative metabolites were generated by performing *Find by Formula* function integrated into the software. The abundance of the EICs was calculated by summing the intensities of all compound-related peaks (e.g. isotopic peaks, adduct peaks, etc.). The pre-processed data files were imported into Agilent Mass Profiler Professional software (version 12.1) for further statistical analysis. MS/MS spectra and retention times acquired from reference metabolites were used for confirmation of the identification of statistically significant metabolites. More specifically, the exact m/z values and intensities of fragment ions from the acquired MS/MS spectra of putative metabolites must have a reasonable match with that of reference metabolites or the fragment ions from public databases (e.g. METLIN, MassBank), if available.

### Statistical Data Analysis

To identify statistical significant differences between the control and IUGR fetal brain tissue samples the processed data files were imported into Agilent Mass Profiler Professional software version 12.1 (Agilent Technologies, Santa Clara, CA) for statistical analysis. The intensities of the 78 identified metabolites in control and IUGR brain tissue samples were compared using a t-test (cut-off p<0.05) followed by a Benjamini-Hochberg Multiple Testing Correction. To visualize the variation within the dataset and most contributing variables (metabolites), the significant metabolites were used to perform a Principal Component Analysis (PCA) using the first three components for data classification. A hierarchical cluster analysis was used to demonstrate if control and IUGR fetal brain tissue samples could be distinguished based on the profile of the significantly altered metabolites. Correlations between fetal birth weight and metabolite intensities were performed in Graphpad Prism version 5 (GraphPad software, San Diego, CA).

## Results

### Fetal Birth and Brain Weight of Control and IUGR Rabbit Fetuses

In total nine living (five control and four IUGR) and seven stillborn (one control and six IUGR) fetuses were collected. The mortality rate (60%) in the group of IUGR fetuses is inherent to the induced IUGR conditions and in accordance with previous studies [24]. The fetal birth and brain weight of the IUGR rabbit fetuses was significantly lower compared to the control fetuses (Table 1). The average birth weight for control fetuses (n = 5) was 52.73±0.53 g and for IUGR (n = 4) 33.86±7.17 g. The average brain weight was 1.32±0.05 g for control and 1.07±0.13 g for IUGR fetuses. One of the selected IUGR fetuses (I) had a relatively high birth weight of 44.54 grams, which suggests it developed under less severe IUGR conditions compared to the other three IUGR fetuses.

**Table 1:** The birth and brain weight of the collected control and IUGR rabbit fetuses.

Control			IUGR		
Fetus	Birth weight (g)	Brain weight (g)	Fetus	Birth weight (g)	Brain weight (g)
A	52.57	1.33	F	29.22	0.92
B	52.63	1.36	G	31.22	1.14
C	52.53	1.35	H	30.46	1.01
D	53.63	1.25	I	44.54	1.19
E	52.27	1.32			
n ± SD	52.73±0.52	1.32±0.05	n ± SD	33.86±7.17 (***)	1.07±0.13 (**)

Data are presented as mean with standard deviation.

** denotes a statistically significant difference with p<0.01, and *** with p<0.001.

### Metabolic Differences between Control and IUGR Fetal Brain Tissue

**Figure 1:**
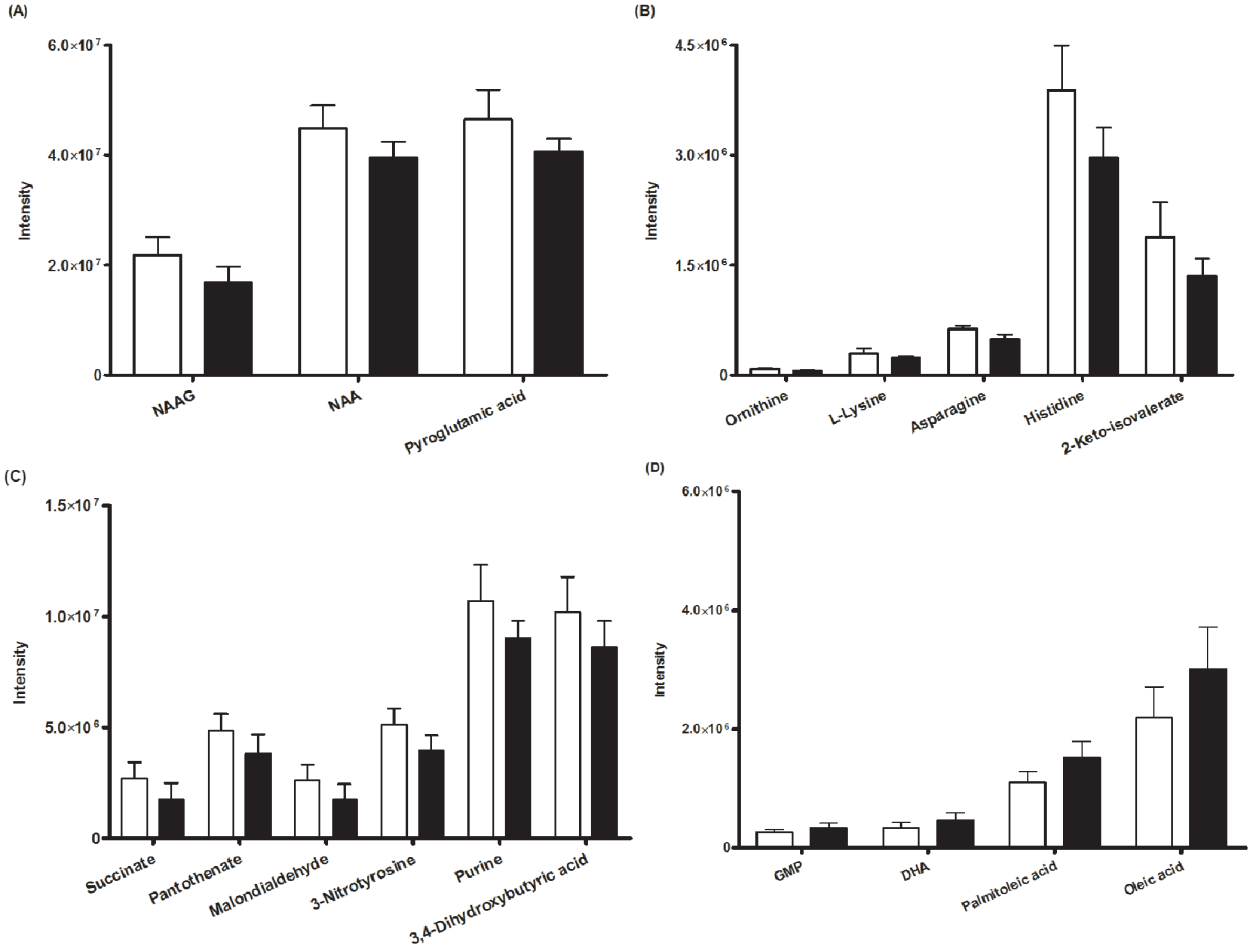
Metabolic differences between control and IUGR fetal brain tissue. Bar charts show the measured metabolite ion intensities of NAAG, NAA, pyroglutamic acid (A), ornithine, L-lysine, aspargine, histidine and 2-keto-isovalerate (B), succinate, panthothenate, malondialdehyde, 3-nitrotyrosine, purine and 3,4-Dihydroxybutyric acid (C), GMP, DHA, palmitoleic acid and oleic acid (D) in control (white bars) and IUGR (black bars) fetal brain tissue. All metabolite ion intensities were significantly different (p<0.05) based on a t-test followed by a Benjamini-Hochberg Multiple Testing Correction. Data are presented as mean, control (n = 5) and IUGR (n = 4) fetal brains with error bars representing standard deviation.

The matching of m/z values and extracted ion chromatograms against the metabolite database and subsequent manual check for matching scores and peak shapes resulted in the identification of 78 metabolites. To investigate if IUGR leads to metabolic differences in the fetal rabbit brain, the ion intensities of the 78 metabolites in the control and IUGR fetal brain tissue samples were compared using a t-test. Results showed that 18 of the 78 metabolites were significantly different (p<0.05). The statistically significant metabolites, their p-values and description are shown in Table 2. To visualize the metabolic differences, the mean metabolite intensities in the control and IUGR fetal brain tissue samples were plotted in graphs (Fig. 1A–D). The graphs showed that most metabolites were significantly decreased in the IUGR fetal brain tissue samples, including the brain metabolites; N-acetylaspartylglutamic acid (NAAG), N-acetylaspartate (NAA) and pyroglutamic acid, (Fig. 1A), the amino acids; ornithine, L-lysine, aspargine, histidine and leucine intermediate 2-keto-isovalerate, (Fig. 1B), the energy metabolism intermediates; succinate and panthothenate, oxidative stress metabolites malondialdehyde and 3-nitrotyrosine, the nitrogen containing heterocycle purine and organic acid 3,4-dihydroxybutyric acid (Fig. 1C). Metabolites that were significantly increased included the nucleotide GMP and unsaturated fatty acids; docosahexaenoic acid (DHA), palmitoleic acid and oleic acid (Fig. 1D).

**Table 2:** Metabolites with significantly different ion intensities in control and IUGR fetal brain tissue.

Metabolite	P value	Mass	Description
Asparagine	0,0000242	132,0534	Amino acid
Ornithine	0,0000275	132,0894	Amino acid
Palmitoleic acid	0,0000372	254,2249	Monounsaturated fatty acid
3-Nitrotyrosine	0,000543	226,0599	Marker of protein nitration
N-Acetylaspartylglutamic acid (NAAG)	0,000632	304,0914	Neurotransmitter
2-keto-isovalerate	0,001535	116,0474	Leucine biosynthesis intermediate
GMP	0,001535	363,0578	Nucleotide
Histidine	0,002027	155,0695	Amino acid
Docosahexaenoic acid (DHA)	0,002243	328,2408	Polyunsaturated fatty acid
N-acetylaspartate (NAA)	0,002243	175,0482	Neuropeptide
Purine	0,002375	120,0426	Nitrogen containing heterocycle
Oleic acid	0,003172	282,2564	Monounsaturated fatty acid
L- Lysine	0,003892	146,1056	Amino acid
Pyroglutamic acid	0,003892	129,0428	Precursor of glutamate
Pantothenate	0,003892	219,1108	Precursor of coenzyme A
Malondialdehyde	0,004639	72,02118	Lipid peroxidation marker
Succinate	0,005183	118,0267	Citric acid cycle intermediate
3,4-Dihydroxybutyric acid	0,029617	120,0425	Organic acid

According to a t-test followed by a Benjamini-Hochberg Multiple Testing Correction.

### Principal Component and Hierarchical Cluster Analysis

**Figure 2:**
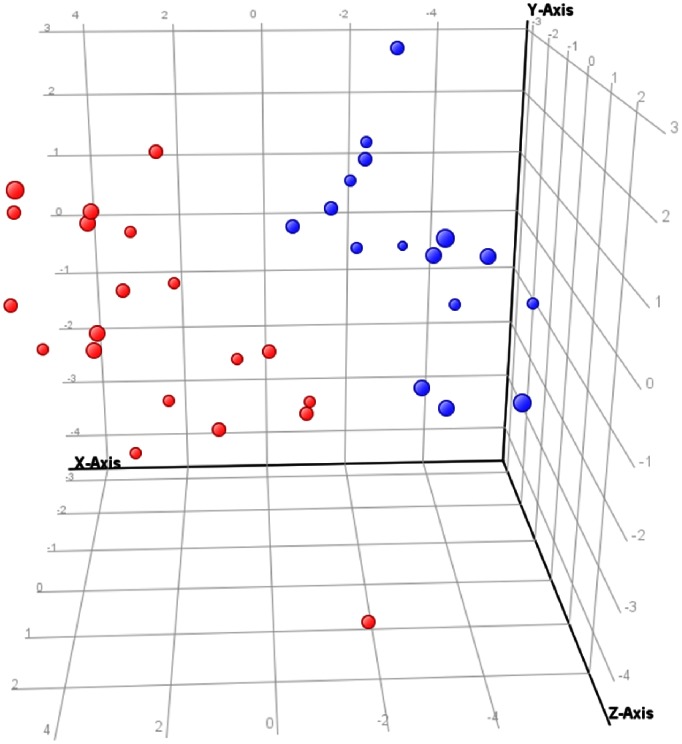
Principal Component Analysis (PCA) score plot showed a clear cluster separation of the control (blue) and IUGR (red) fetal brain tissue samples. The PCA was based on the significant metabolites identified by the t-test. The plot shows the first three principal components that include PC1∶49.83%, PC2∶12.77% and PC3∶9.23% percentage of total variables.

**Figure 3:**
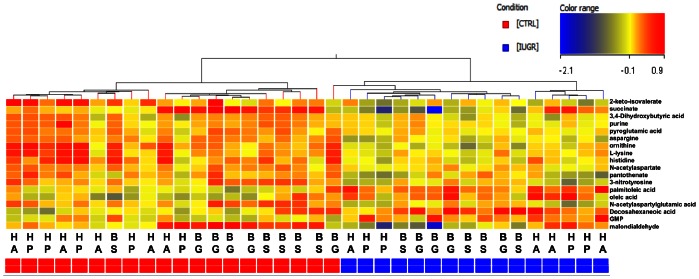
Dendrogram and heatmap showing the metabolic differences between control and IUGR fetal brain tissue samples. The significantly altered metabolites were hierarchically clustered (columns) and ratios plotted on a color scale (rows). The dendrogram showed a clear cluster formation between the control (blue) and IUGR (red) samples. In addition, smaller clusters are visible, which mostly group the hemisphere anterior (HA), hemisphere posterior (HP) basal ganglia (BG) and brain stem (BS) regions.

To study the statistically significant metabolic alterations in the fetal brain due to the induction of IUGR more in depth, both a principal component and hierarchical cluster analysis were performed based on the ion intensities of the 18 significant metabolites in the control and IUGR fetal brain tissue samples. The results of the PCA (Fig. 2) showed that the control (in blue) and IUGR (in red) fetal brain samples were clearly separated in two groups along the first three principal components of the score plot. The corresponding X-loading values (Table 3) revealed that the metabolites, aspargine, NAA, pyroglutamic acid (positive correlation) and palmitoleic acid (negative correlation) largely contributed to the cluster formation along the first principal component. The resulting heat map and dendrogram of the hierarchical cluster analysis (Fig. 3) also showed two main clusters that separated the control and IUGR fetal brain tissue samples. In addition both clusters were subdivided in smaller clusters, which largely reflected the different brain regions analyzed, creating groups of hemisphere, basal ganglia and brain stem tissue samples. Therefore the obtained results indicated that each brain region comprises a characteristic metabolite profile and that the induction of IUGR caused metabolic alterations in all brain regions. In general the PCA and hierarchical cluster analysis revealed the potential to distinguish between the normal and IUGR fetal brain based on the comprehensive measurement of its metabolic profile or status.

**Table 3:** The X-loading values for the first principal component of the PCA score plot.

Metabolite	PC1 (49.83%) value
Asparagine	0.290
NAA	0.286
Pyroglutamic acid	0.283
Ornithine	0.273
3-Nitrotyrosine	0.272
NAAG	0.271
L-Lysine	0.269
Purine	0.259
Histidine	0.258
3,4-Dihydroxybutyric acid	0.248
2-Keto-isovalerate	0.239
Pantothenate	0.237
Malondialdehyde	0.166
Succinate	0.162
Oleic acid	−0.135
GMP	−0.147
Docosahexaenoic acid	−0.155
Palmitoleic acid	−0.183

X-loading values show positive and negative correlations responsible for the cluster formation along the first principal component (PC1) in the PCA score plot.

### Correlations between Fetal Birth Weight and Metabolite Intensity Alterations

**Figure 4:**
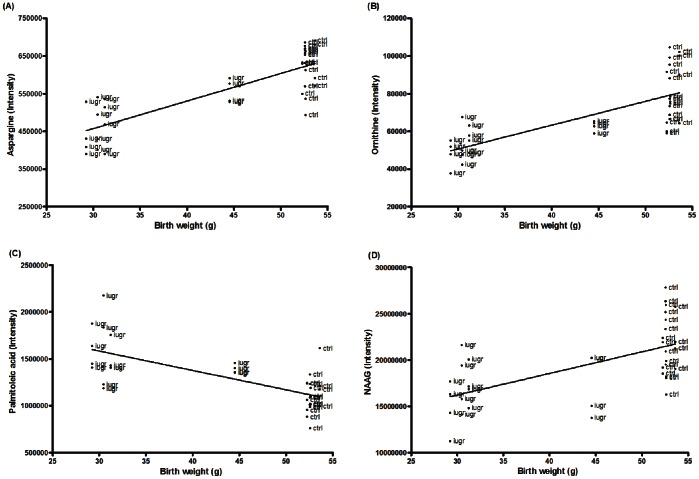
Correlation graphs between fetal birth weight and metabolite ion intensities. The metabolites aspargine (r^2^ = 0.6723) (A), ornithine (r^2^ = 0.5422) (B), palmitoleic acid (r^2^ = 0.4938) (C) and NAAG (r^2^ = 0.3712) (D) showed the highest correlation coefficient. Brain tissue samples from IUGR fetus with the highest birth weight (I), showed metabolite intensities more similar to the controls. Each point represents a single fetal brain tissue sample.

In the clinical setting, estimated fetal birth weight is used as a predictor for the severity of IUGR conditions. To investigate a potential relationship between fetal birth weight and metabolic alterations in the fetal brain of a rabbit IUGR model, the birth weight of the control and IUGR fetuses was correlated with the intensities of the 18 significantly altered metabolites. As expected, the results showed a negative relationship for the decreased metabolites and positive relationship for the metabolites that were increased in the fetal IUGR brain. The highest correlation coefficients between birth weight and metabolite intensities were observed for the metabolites aspargine (r^2^ = 0.6723), (Fig. 4A), ornithine (r^2^ = 0.5422) (Fig. 4B), palmitoleic acid (r^2^ = 0.4938), (Fig. 4C) and NAAG (r^2^ = 0.3712) (Fig. 4D). The correlations also revealed that the brain tissue samples obtained from the IUGR fetus with the highest birth weight (fetus I; 44.45 g) included metabolite ion intensities more similar to the controls (Fig. 4A–D). This suggests that the observed alterations in metabolite ion intensities were dependent on the severity of the IUGR conditions, as reflected by fetal birth weight.

## Discussion

The present study applied a targeted metabolomics approach in a rabbit IUGR model to obtain improved insight into the effects of IUGR on the developing fetal brain and identify potential metabolite candidates to develop perinatal biomarkers for IUGR related abnormal neurodevelopment. Results showed that the induction of IUGR by 40–50% uteroplacental vessels ligation in the pregnant rabbit caused a significant decrease in the fetal birth and brain weight of the rabbit fetuses. These findings correspond with clinical observations in IUGR neonates, who besides a low birth weight were shown to have a decreased brain volume [27]. Therefore this study provides further supporting evidence that the rabbit IUGR model used is able to reproduce physiological features of the human IUGR condition and therefore could provide clinically relevant information regarding the effects of IUGR on fetal brain. Targeted metabolic profiling in the rabbit IUGR model showed that IUGR leads to statistically significant metabolic alterations in the fetal rabbit brain. The metabolic differences included ion intensity alterations in neurotransmitters/peptides, amino acids, fatty acids, energy metabolism intermediates and oxidative stress related metabolites. The identified alterations in brain metabolites could be useful to support the development of clinical biomarkers for the early perinatal diagnosis of IUGR related abnormal neurodevelopment and the discovery of potential therapeutic targets for fetal therapy.

Previous studies in animal model of the IUGR condition have shown that IUGR adversely affects brain development and organization [5]–[6]. The results obtained in this study showed a statistically significant decrease in the brain specific metabolites NAAG and NAA in the brain tissue of IUGR fetuses compared to control fetuses. NAAG and NAA are primarily present in neurons and oligodendrocytes and are well known markers for neuronal density and viability [28]. Moreover, NAA is synthesized in neuronal mitochondria and is therefore considered a marker for neuronal mitochondrial metabolism [29]. The observed decrease in NAAG and NAA metabolites therefore indicate that IUGR has a direct adverse effect on neurons and oligodendrocytes by perturbing neuronal viability, energy metabolism and the myelination process. These results are in agreement with previous brain histology studies in animal IUGR models, which demonstrated that IUGR adversely affected the myelination process [30] and caused the induction of neuronal cell death [6], [31]. Moreover, a previous MRI study in the identical rabbit IUGR model observed a decrease in white matter myelination [26]. A potential adverse effect of IUGR on neuronal energy metabolism was supported by the identified decrease in the energy metabolism intermediates succinate and pantothetic acid. Decreased succinate levels have been previously proposed as an indicative marker for disrupted neuronal energy metabolism in an Alzheimer's disease mice model [32]. A study of brain metabolism in normal and IUGR fetuses using proton magnetic resonance spectroscopy also showed decreased NAA levels in the fetal IUGR brain [33]. Therefore the identified decrease in NAAG and NAA metabolites in the rabbit IUGR model further support previous findings that IUGR alters neuronal metabolism in the fetal brain.

Besides neurospecific metabolites, results showed a statistically significant decrease in the ion intensities of several amino acids in the fetal IUGR brain, including aspargine, histidine, ornithine, L-Lysine. Previous studies in animal models have already demonstrated that IUGR due to placental insufficiency causes a reduced placental transport of amino acids to the fetal blood circulation [33]–[35]. In addition, measurements of amino acids concentrations in maternal and fetal blood plasma of normal and IUGR pregnancies showed a significant reduction in amino acid levels in the blood plasma of IUGR fetuses [36]. The results obtained in this study suggest that IUGR can lead to reduced amino acid levels in the fetal brain of a rabbit model. Because amino acids are important for brain growth, metabolism and function, a potential reduction in the fetal brain could have a role in IUGR related abnormal neurodevelopment such as changes in brain volumes [27], [37], connectivity patterns [9] and cognitive dysfunctions [11]–[12].

Metabolites found to be statistically significant increased in the IUGR fetal rabbit brain included the monounsaturated fatty acids palmitoleic acid, oleic acid and polyunsaturated fatty acid DHA. Both types of fatty acids are essential parts of brain cell membranes and myelin sheets and play a crucial role in neurodevelopment [38]–[40]. From animal and clinical studies it is known that fatty acids accumulate during pregnancy in the adipose tissue and brain of the developing fetus [41]–[42]. There have been few studies on brain fatty acid profiles in animal models of IUGR, one reported study in a rat IUGR model found no significant differences in fatty acid composition between the control and IUGR fetal brain [43]. In clinical studies however, it was shown that IUGR fetuses have higher levels of free fatty acids and triglycerides in blood plasma compared to normal fetuses, related to changes in lipid metabolism and distribution [44]. Further studies on fetal brain lipid compositions in normal and IUGR pregnancies should clarify if the increased lipid levels detected in the rabbit model are clinically relevant.

Metabolic profiling also showed alterations in oxidative stress related metabolites including the lipid peroxidation marker malondialdehyde [45] and protein nitration marker 3-nitrotyrosine [46]. Although IUGR has been mostly shown to induce an oxidative stress response, both markers were found to be decreased in the fetal IUGR brain tissue. Since the redox status of a biological system changes rapidly, the levels of oxidative stress metabolites depend largely on the time point of measurement. It is likely that the release of hypoxic-ischemic conditions after delivery by cesarean section changed the dynamic oxidative stress related metabolism in the fetal brain. Therefore, it is difficult to interpret the measured alterations in the oxidative stress markers and draw any conclusions. Nevertheless the fact that both oxidative stress markers were among the significantly altered metabolites suggest that oxidative stress plays a role in the fetal brain under IUGR conditions. An improved study design allowing the *in vivo* measurement of oxidative markers in the fetal brain of the rabbit IUGR model would be required to obtain a detailed understanding of the oxidative stress response in the IUGR fetal brain.

Multivariate data analysis by PCA and hierarchical cluster analysis demonstrated that control and IUGR fetal brain tissue samples (from the identical pregnant rabbit) could be clearly distinguished based on their metabolite profiles. The PCA loadings indicated that the neuropeptide (NAA) amino acid (aspargine, ornithine) and fatty acid (palmitoleic acid) primarily contributed to the separation along the first principal component, which included most of the total variance in the dataset. These contributions make these metabolites interesting candidates for the development of clinical biomarkers using non-invasive clinical imaging techniques in the fetal brain. Even though the PCA results showed that IUGR had a global effect on the fetal rabbit brain by separating control and IUGR brain tissue samples from all studied brain regions, the metabolic differences were found to be predominant in the hemisphere brain regions. This suggests that the frontal brain regions are primarily susceptible for IUGR conditions, as was earlier suggested by clinical imaging [47] and behavioral studies [48].

### Strengths and Limitations

The main strength of this study includes the unique combination of a rabbit IUGR model and metabolomics approach. The rabbit IUGR model was previously shown to mimic the human IUGR condition. Furthermore it enables a direct comparison of control and IUGR fetuses from the same mother. This study design reduces variability factors and avoids the requirements of large study groups of animals. Metabolomics can provide information on phenotypic outcomes of disease processes (IUGR conditions), which are highly relevant for biomarker candidate identifications. The sensitivity of the LC-QTOF-MS metabolomics technology allowed the detection of metabolite alterations related to subtle effects of IUGR conditions on the fetal brain. Limitations include the quantification of metabolites in brain tissue extracts instead of the tissue itself, therefore the measured metabolite levels might not correspond with *in vivo* concentrations. To overcome this limitation the *in vivo* metabolite levels should be determined by non-invasive techniques such as NMR or MRI spectroscopy. Nevertheless these techniques do not have the sensitivity of the applied LC-QTOF-MS approach. As expected not all measured metabolites could be identified using the current up-to-date metabolite databases. Therefore there could be additional metabolite candidates suitable for clinical biomarker development. The raw data of this study will be stored and as metabolite databases will expand there will be opportunities for additional metabolite identifications. Finally this study was only able to provide a snapshot view of the dynamic metabolic processes in the fetal IUGR brain. To have a more comprehensive understanding of the progressive adverse effects of IUGR on the fetal brain, a larger scale time course metabolomics study would be required. Such a comprehensive analysis was beyond the scope of this initial study; however the results here obtained encourage the performance of an extended metabolomic profiling study. Despite these limitations the results of this study provide new mechanistic insights and demonstrate the potential to develop a prediction model for the effects of IUGR on the fetal brain based on tissue metabolic profiles.

From a clinical perspective, this study provides preliminary evidence supporting further research on the development of metabolic biomarkers associated with growth restriction and its neurodevelopmental correlates. Current challenges in fetal medicine include the correct classification of true growth restriction from physiologic smallness, particularly among fetuses with borderline estimated fetal weights. Such differentiation is critical to establish early-life intervention measures to correct for the impact of IUGR on neurodevelopment. Thus, it is imperative to develop classification algorithms. Likewise, biomarkers will be a critical need for the monitoring of the effectiveness of interventions in the correction of neurodevelopmental deviations. The design of such models will necessarily integrate clinical parameters, such as fetal birth weight and hemodynamic measures, together with more sophisticated blood or imaging biomarkers. Brain metabolic signatures could be determined by MRI spectroscopy and be useful markers as part of predictive scores to facilitate the perinatal diagnosis and monitoring of IUGR related abnormal neurodevelopment. Implementation of metabolomics technologies in clinical practice will require translational studies to demonstrate the feasibility of measuring metabolite candidates by means of non-invasive clinical imaging techniques in animal models and eventually patients.

In summary, this targeted metabolomics study demonstrates that IUGR leads to statistically significant metabolic alterations in the fetal rabbit brain, involving neuronal viability, energy metabolism, amino acid levels, fatty acid profiles and oxidative stress mechanisms. The identified metabolic alterations showed a dependence on the severity of IUGR conditions (reflected by birth weight) and allowed a clear discrimination between control and IUGR fetal brain tissue, which revealed the potential to develop biochemical biomarkers for the perinatal diagnosis of IUGR at its related abnormal neurodevelopment. Overall results identified aspargine, ornithine, NAAG, NAA and palmitoleic acid as the most promising metabolite candidates for further biomarker development studies based on non-invasive clinical imaging techniques.
